# Patient-specific three-dimensional explant spheroids derived from human nasal airway epithelium: a simple methodological approach for ex vivo studies of primary ciliary dyskinesia

**DOI:** 10.1186/s13630-017-0049-5

**Published:** 2017-03-23

**Authors:** June Kehlet Marthin, Elizabeth Munkebjerg Stevens, Lars Allan Larsen, Søren Tvorup Christensen, Kim Gjerum Nielsen

**Affiliations:** 1 Danish PCD & chILD Centre, CF Centre Copenhagen, Paediatric Pulmonary Service, Department of Paediatrics and Adolescent Medicine, Copenhagen University Hospital, Rigshospitalet, Blegdamsvej 9, Copenhagen, DK-2100 Denmark; 2 Wilhelm Johannsen Centre for Functional Genome Research, Department of Cellular and Molecular Medicine, University of Copenhagen, Blegdamsvej 3, DK-2200 Copenhagen, Denmark; 3 Department of Biology, Section of Cell Biology and Physiology, University of Copenhagen, Universitetsparken 13, DK-2100 Copenhagen, Denmark

**Keywords:** Airway epithelial, Cell culture, Cilia, ciliogenesis, Three-dimensional explant spheroids, Primary ciliary dyskinesia, Ciliary beat pattern, Ciliary beat frequency, Diagnosis

## Abstract

**Background:**

Three-dimensional explant spheroid formation is an ex vivo technique previously used in studies of airway epithelial ion and water transport. Explanted cells and sheets of nasal epithelium form fully differentiated spheroids enclosing a partly fluid-filled lumen with the ciliated apical surface facing the outside and accessible for analysis of ciliary function.

**Methods:**

We performed a two-group comparison study of ciliary beat pattern and ciliary beat frequency in spheroids derived from nasal airway epithelium in patients with primary ciliary dyskinesia (PCD) and in healthy controls. Nasal ciliary cells and sheets were removed on day 1 by nasal brush biopsy and analyzed with regard to ciliary beat pattern—and frequency using high-speed video imaging for standard reference values. Three-dimensional explant spheroid formation was initiated in the same individual on the same day by incubation of cells and sheets from a separate brush biopsy. Harvested spheroids were analyzed earliest possible and values of spheroid ciliary beat pattern and frequency were compared to the corresponding reference values from day 1.

**Results:**

Spheroids formed fast in serum-free culture medium. Formation was successful in 15 out of 18 (82%) sampled individuals. Thus, formation was successful in seven healthy controls and eight PCD patients, while unsuccessful in 3 with PCD due to infection. Median (range) number of days in culture before harvesting of spheroids was 4 (1–5) in healthy versus 2 (1–5) in PCD. Spheroid ciliary beat pattern and frequency were unchanged compared to their corresponding day 1 standard reference values. Spheroid ciliary beat frequency discriminated highly significant between healthy controls (9.3 Hz) and PCD patients (2.4 Hz) (*P* < 0.0001). Survival of spheroids was 16 days in a single healthy person.

**Conclusion:**

Patient-specific three-dimensional explant spheroid formation from a minimal invasive nasal brush biopsy is a feasible, fast and valid ex vivo method to assess ciliary function with potential of aiding the diagnosis of PCD. In addition, it may be a useful model in the investigation of pathophysiological aspects and drug effects in human nasal airway epithelium.

**Electronic supplementary material:**

The online version of this article (doi:10.1186/s13630-017-0049-5) contains supplementary material, which is available to authorized users.

## Background

Primary ciliary dyskinesia (PCD) is a rare, ciliopathic, autosomal recessive or X-linked inherited genetic disorder that causes defects in the movement of cilia lining the respiratory tract, leading to deficient mucociliary clearance and severe, recurrent or chronic upper and lower respiratory infections. Diagnosis of PCD is complicated, requiring advanced equipment and a high degree of expertise. According to the new ERS task force guideline for the diagnosis of PCD, it is recommended to improve the diagnostic accuracy of high-speed video analysis (HSVA) findings by repeating assessments of ciliary beat pattern (CBP) and ciliary beat frequency (CBF) after cell culture as part of a step-wise work-up algorithm [1].

The steps include:

Step 1: Measurement of nasal nitric oxide (nNO) and performing HSVA of ciliated cells removed from the nose or lung and placed under a microscope for analysis of CBP and CBF immediately after biopsy.

Step 2: Investigation of ciliary ultrastructural defects by transmission electron microscopy (TEM) and repeating HSVA after cell culture. Normal TEM does not rule out PCD.

Step 3: Genetic testing. There are more than 35 known PCD-causing genes. Negative genetic testing does not rule out PCD.

Assessment of moving cilia in cultured cells, as means to assist PCD diagnosis, has been described as “the ciliogenesis principle”. In this procedure, a two-dimensional primary culture of ciliated cells is expanded and grown on collagen-coated plastic dishes until a confluent monolayer is reached, and during which the cells are deciliated. Following this procedure, re-growth of new cilia can be induced, either in suspension [2, 3] or on air liquid interface (ALI) [4, 5].

Ciliogenesis requires approximately 3–6 weeks from biopsy before new cilia are fully re-grown and ciliary movement can be analyzed [6].

By re-testing ciliary function and/or ultrastructure in newly differentiated cilia after ciliogenesis, secondary ciliary defects are intentionally avoided, which can aid either the confirmation or exclusion of the PCD diagnosis in cases where the initial investigation has been difficult [6–8]. In addition to aid difficult PCD diagnosis, in vitro culturing of airway epithelial cells in general serves the purpose of investigating a wide range of physiological parameters associated with the airway epithelium.

In this study, we present a third model for assessing ciliary cells in culture.

Patient-specific three-dimensional explant (3D-E) monolayered spheroid formation is a minimal invasive, simple and fast ex vivo method to assess ciliated airway cells from the nose. Nasal epithelial cells are removed by a nasal brush biopsy and placed directly in culture medium, in which they spontaneously form 3D-E spheroids without undergoing the processes of ciliogenesis. 3D-E spheroids are accessible for analysis of ciliary function within only a few days.

Our method comprises a modified version of the previous well-described model by Pedersen and colleagues, where production of free-floating fluid-filled three-dimensional airway epithelial spheroids derived from protease released epithelium of resected human nasal polyps was performed [9–12]. Their spheroids could be assessed already a few days after polyp resection and have been used for extensive basic research, investigating water permeability and ion transport in healthy and cystic fibrosis airway epithelium [9–12].

We here present a simple and rapid method to produce ex vivo 3D-E spheroids that can be used to discriminate between PCD patients and healthy controls by differences in CBP and CBF. Feasibility, validity and success rates of the method were included in the study. Part of this study has been previously presented as a poster at an American Thoracic Society Conference [13].

## Methods

### Participants

Healthy non-smoking individuals and patients with confirmed PCD diagnosis were included after written informed consent.

### PCD patients recruited for the study

Definite PCD patients were recruited from the National Danish PCD cohort in whom cystic fibrosis and immunodeficiency had been excluded. PCD diagnosis was based on characteristic clinical symptoms in combination with at least twice repeated high-speed video-microscopic recordings showing abnormal ciliary beat pattern and frequency. Additional PCD work-up included evaluation by TEM analysis, nasal nitric oxide, immunofluorescence (IF) staining and genetic testing for specific PCD mutations. For TEM analysis, at least 100 ciliary cross-sections per patient were evaluated for ultrastructural defects. Nasal nitric oxide (NO) measurements were performed using NIOX^®^ (nitric oxide monitoring system; Aerocrine, Solna, Sweden) equipment. Gas was aspirated via a nasal olive probe inserted into one nostril by use of passive sampling flow rate of 5 mL s^−1^ (~0.3 L min^−1^). All PCD patients in this study cooperated to nasal NO breath-hold maneuver. A cut off value of 175 ppb was used in alignment with our previous established reference for nasal NO breath-hold sampling [14]. IF staining and genetic testing were performed post-hoc in some of the PCD patients participating in the current study, as part of a general ongoing evaluation of our Danish PCD cohort. These tests were performed at The Research Laboratory of Prof. Heymut Omran, University Hospital Muenster, Department of General Paediatrics, Muenster, Germany.

### Nasal brush biopsy

Nasal ciliary cell sheets were removed by nasal brush biopsies from the upper third part of the nose, one biopsy from each nostril. One biopsy was used for baseline conventional ciliary function analysis (cCFA) using high-speed video recordings and one was used for the 3D-E spheroid formation.

### Conventional ciliary function analysis

Ciliary beat pattern and CBF in 5–10 nasal epithelial strips were analyzed at room temperature immediately after removal from the nose by use of an interference contrast microscope (Leica DMLB; Leitz, Stuttgart, Germany) and by digital frame-by-frame assessment of ciliary movement and HSVA. Digital image sampling was performed at 200 frames per second. cCFA was used as our standard reference method.

## 3D-E spheroid formation

3D-E spheroid formation followed upon incubation of cells and cell sheets from a simple nasal brush biopsy as described for cCFA. The nasal material adherent to the brush was released by gently swirling the brush directly into a 10-mL Falcon tube containing 37 °C DMEM:HAM-F12 (1:1) medium, supplemented with a serum substitute (1% Ultroser-G, IBF Biotechnics, Savage, MD, USA), antibiotics (105 UL^−1^ of penicillin, 100 mgL^−1^ of streptomycin) and 2 mM l-glutamine. The cell material was then transferred to 2 wells in a 24-well plate (un-coated) and further supplemented with culture medium up to 2/3 of the well volume to prevent the cells from drying out. The well plate was placed in a CO_2_ incubator (5% CO_2_, 37 °C), and during the first 4 h of incubation, the well plate was tapped gently every 15 min to prevent attachment of the epithelial cells to the bottom of the wells. The culture medium was changed daily for the first 2 days and three times a week thereafter by simple sedimentation of cell material for 15 min in a test tube.

Once the spheroids had formed, a single spheroid would be picked randomly from the well by a pipette for analysis. To assess CBP and CBF, the spheroids were immobilized, between an object glass and a cover glass, leaving the spheroids slightly flattened but with preserved ciliary motility, which was easily measured using the same video microscope under the same technical conditions as described for cCFA (HSVA). Following CBP and CBF analysis, the spheroids were killed. The remaining spheroids were left alive in the wells for future analysis by changing the medium as described above.

### Fluorescence microscopy analysis

Suspended samples of epithelial cells from brush biopsies were spread onto cover slides, air dried, and stored at −80 °C until use as previously described [15, 16]. Cells on defrosted slides were fixed in 4% PFA and subjected to differential interference contrast (DIC) microscopy and immunocytochemical (ICC) analysis [17] with antibodies directed against acetylated α-tubulin (mouse anti Ac-Tub; Sigma Aldrich), glutamylated α-tubulin (rabbit anti Glu-Tub; Sigma Aldrich). For immunohistochemical (IHC) analysis, 3D-E spheroids in culture medium were transferred to Falcon tubes and pelleted at ca. 150×*g* followed by fixation in 4% PFA for 15 min. Fixed spheroids were isolated by centrifugation and washed with PBS followed by addition of 70% ethanol, dehydration, paraffin embedding and sectioning at a thickness of 5 µm, and sections were analyzed by DIC microscopy and IHC as previously described [18] with Ac-Tub and Glu-Tub antibodies. Cells and sections were incubated in corresponding Alexa Flour^®^-conjugated secondary antibodies (Invitrogen) for 45 min at RT, and cell nuclei were labeled with DAPI staining. Images were captured on a fully motorized Olympus BX63 upright microscope with a DP72 color, 12.8 megapixel, 4140 × 3096 resolution camera.

### Statistical analysis

Paired *t* test was used for changes in CBP and CBF between cCFA values and 3D-E values for the same individual. Unpaired *t* test was used for two-group comparisons of CBP and CBF in 3D-E spheroids from PCD patients versus healthy controls.

## Results

**Fig. 1:**
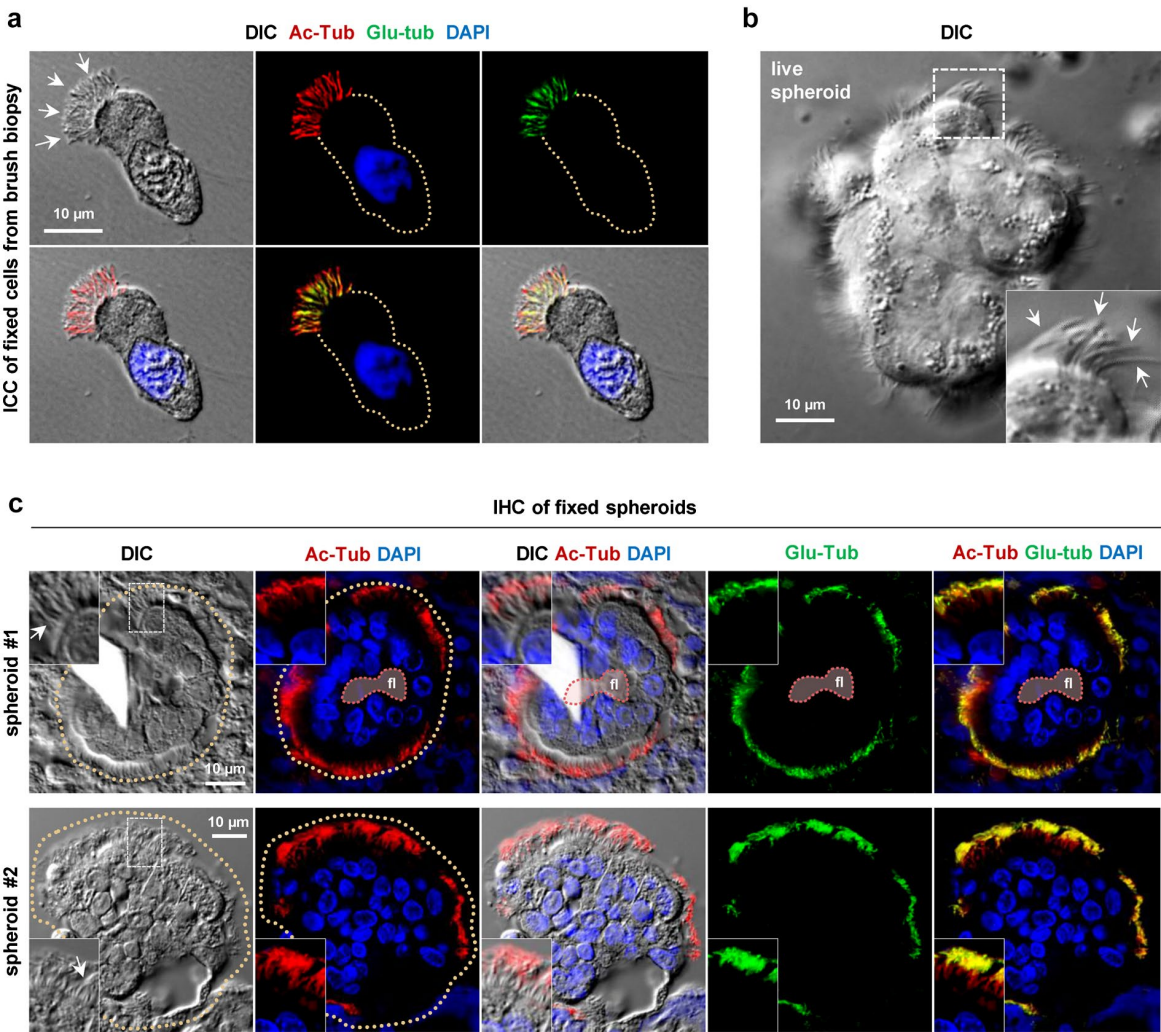
Immunofluorescence microscopy analysis of 3D-E spheroids. **a** Immunocytochemical (ICC) analysis of a ciliated epithelial cell isolated from the nasal brush biopsy. Cilia (*arrows*) are visualized with differential interference contrast (DIC) and with antibodies directed against acetylated α-tubulin (Ac-Tub; *red*) and glutamylated α-tubulin (Glu-Tub; *green*). The nucleus is stained with DAPI (*blue*) and the circumference of the cell is marked with a *dotted line*. **b** DIC microscopy of a live 3D-E spheroid. Cilia are marked with *arrows* (*inset*). **c** Immunohistochemical (IHC) analysis of two 3D-E spheroids (#1 and #2) with DIC and with antibodies and staining reagents as indicated for **a**. Cilia are marked with *arrows* and the circumferences of the spheroids are marked with *dotted lines*. *fl* partly fluid-filled lumen

We included 18 individuals in this proof-of-concept method study: 11 PCD patients and 7 healthy controls. Following nasal brush biopsy, the isolation of epithelial cells was initially confirmed by ICC analysis, showing individual cells ciliated at the apical membrane as judged by DIC and staining with cilia-specific antibodies (Fig. 1a). 3D-E spheroids formed easily and quickly from nasal brush biopsy material when incubated in Ultroser G-supplemented culture medium, and spheroid formation occurred equally quickly in both PCD patients and healthy controls. Ready-for-analysis 3D-E spheroids were obtained with median (range) number of 2 (1–5) days for PCD patients, and 4 (1–5) days for healthy controls (Fig. 1b). Sections of fixed 3D-E spheroids from a healthy donor were further analyzed by IHC analysis, displaying well-organized arrangements of monolayer cells enclosing a partly fluid-filled lumen, with the ciliated apical surface facing the outside (Fig. 1c, upper panels). In some cases, the fluid-filled lumen was undetectable due to sectioning outside this lumen (Fig. 1c, lower panels). In all experiments, the 3D-E spheroids were often interspersed with single cells and other cell materials as a consequence of fixation and centrifugation of the cultures prior to paraffin embedding.

**Fig. 2:**
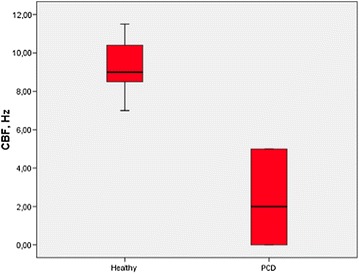
Patient-specific 3D-E spheroid ciliary beat frequency (CBF) measured in beat per second (Hz) in-patients with primary ciliary dyskinesia (PCD) and healthy controls

We found a highly consistent and significant difference in mean CBF values between 3D-E spheroids derived from healthy controls (9.2 Hz) and PCD patients (2.6 Hz) (*P* < 0.0001) (Fig. 2). Hence, our 3D-E spheroids appeared equally discriminative between PCD and health as compared to the initial gold standard cCFA. We observed no differences in CBP and CBF between spheroid and baseline cCFA analyses, neither in healthy controls nor PCD patients. For PCD patients, CBP was consistent when comparing initial cCFA and 3D-E spheroids, both for immotile and dysmotile variants of PCD. CBF seemed not to slow down within the tested window of 5 days, suggesting that spheroids after 5 days in culture still had vital and well-functioning cilia (Table 1).

**Table 1:** Comparison of ciliary beat pattern (CBP) and ciliary beat frequency (CBF) in eight patients with primary ciliary dyskinesia (PCD) and seven healthy non-smoking controls measured by conventional ciliary function analysis (cCFA) performed immediately after brush biopsy and after patient-specific 3D-explant spheroid (3D-E) formation

Healthy	Mean CBF, cCFA (Hz)	Mean CBF, 3D-E (Hz)	CBP, cCFA	CBP, 3D-E	No. of days in culture	EM defects	IF defects	PCD mutation	nNO (ppb)
1	10.7	10.0	Synchronous	Synchronous	4	–	–	–	–
2	10.0	9.8	Synchronous	Synchronous	5	–	–	–	–
3	10.0	11.5	Synchronous	Synchronous	4	–	–	–	–
4	8.8	9.0	Synchronous	Synchronous	5	–	–	–	–
5	8.3	8.0	Synchronous	Synchronous	1	–	–	–	–
6	7.8	9.0	Synchronous	Synchronous	3	–	–	–	–
7	5.3	7.0	Synchronous	Synchronous	3	–	–	–	–
PCD									
1	0	0	Immotile	Immotile	5	ODA	DNAH5 (polyclonal) abnormal	Awaiting results	36
2	0	0	Immotile/stiff flickery	Immotile/stiff flickery	2	Radial spoke head defect +IDA defect	GAS8/11 abnormal and DNALI1 (monoclonal) inconclusive	CCDC40	52
3	0	0	Immotile	Immotile	1	ODA	DNAH5 (polyclonal) inconclusive and DNALI1 (LC28,polyclonal) abnormal	Awaiting results	30
4	3.0	4.0	Asynchronous	Asynchronous	2	ODA	DNAH5 (polyclonal) abnormal	Awaiting results	283
5	3.6	5.0	Asynchronous	Asynchronous	2	ODA	DNAH5 (polyclonal) abnormal	Awaiting results	102
6	5.3	5.0	Asynchronous	Asynchronous	5	Normal	None found	Unknown	101
7	3.6	5.0	Asynchronous	Asynchronous	2	Inconclusive	None found	Unknown	–
8	3.0	2.0	Stiff and asynchronous	Stiff and asynchronous	2	Sample poor, not repeated	Not done	Unknown	36

Healthy spheroids presented themselves in two different ways: either as rotating spheroids, where the ciliary movements made the spheroid spin around its own axis, or as spheroids with beating cilia without rotation.

The rotating spheroids usually were quite large, containing many cells and usually surrounded by mucus, whereas healthy non-rotating spheroids were smaller. Examples of healthy rotating, healthy non-rotating and PCD spheroids are given in Additional files 1, 2 and 3.

The overall success rate of spheroid formation was 82% (15/18). Three samples (all from PCD patients) were lost due to infection [success rate healthy controls = 100%, PCD patients = 73% (8/11)]. The yield of spheroids was quite variable, ranging from a few (2–3) up to approximately 20 spheroids per sample. We did not test maximal survival time of the spheroids in this study. However, one sample of beating spheroids was kept for 16 days, after which the spheroids were terminated.

## Discussion

In this study, we presented a simple and fast method to produce ex vivo 3D-E ciliated spheroids derived from nasal airway epithelium. These spheroids are spontaneously formed from terminally differentiated ciliated cells presenting their original cilia. This in sharp contrast to ciliated organoids derived from the ciliogenesis principle, which requires multiple steps of cell culturing involving cell multiplication, differentiation and re-growth of the cilia. The spheroids were further allowed to stay in the culture medium together with the mucus that came along with the brush biopsy, thereby partly mimicking the in vivo situation. No enzymatic splitting or spinning of the cells was performed and the rapid way of forming spheroids allowed them ready for ciliary function testing after only 1–5 days after brush biopsy, as compared to otherwise 3–6 weeks by, e.g. the ALI method [6].

Table 2 sums up the main characteristics and potentials of the three methods: (1) ciliogenesis from suspension, (2) ciliogenesis from ALI and (3) 3D-E spheroids.

**Table 2:** Characteristics and potentials of three airway epithelial culture methods

	Crude immediate brush material [21]	2-step ALI [5, 8, 22, 23]	2-step suspension [2, 3, 24, 25]	3D-E spheroids [9, 13]
Characteristics				
Sampling of cells	Nasal brush biopsy	Nasal brush biopsy	Removal of nasal polyp or nasal forceps biopsy	Removal of nasal polyp/nasal brush biopsy
Anesthesia required	No	No	Yes	Yes (polyp removal) No (nasal brush)
Preparation steps	Cell collection Cell dispersion	Cell collection Cell dispersion Cell proliferation Ciliary regeneration	Tissue washing Enzymatic tissue digestion Cell dispersion Cell proliferation Ciliary regeneration	Cell collection Cell dispersion Spheroid formation
Longevity of cells/culture	1–3 days	>1 month	>1 month	At least 16 days
Time from sampling to analysis	Immediately	4 weeks	4–6 weeks	24 h
Materials and reagents	Falcon tube NaCl Glass slide	Falcon tube Culture medium T25/75 flask 24-well plate Transwell insert filters D-PBS Trypsin–EDTA Trypsin-inhibitor Centrifuge CO_2_ incubator	Falcon tube Pronase Culture medium Plastic plate Collagen gel Collagenase T25/75 flask Gyrotori shaker CO_2_ incubator	Falcon tube Culture medium 24-well plate CO_2_ incubator
Complexity	Simple	Complex	Complex	Simple
Terminally differentiated cells	Yes	No	No	Yes
Cell divisions	No	Yes	Yes	No
Ciliogenesis	No	Yes	Yes	No
Known applications				
Diagnostics	Yes	Yes	Yes	Yes
Study of transport mechanisms	No	Yes	No	Yes
Study of ciliary physiology	Yes	Yes	Yes	Yes
Potential applications				
Study of drug effects	No	Yes	Yes	Yes

Ciliogenesis from suspension, ciliogenesis from air–liquid interface (ALI) and 3D-E spheroids

Compared to previous described primary cell culture techniques for investigating airway ciliated cells, we found 3D-E spheroid formation to be advantageous and promising in a number of ways that may make the method a useful alternative to other primary ciliary cell culture methods:

- Ready-to-test spheroids were produced in only a few days after brush biopsy.
- The sampling technique was minimally invasive. By a simple and quite gentle brush biopsy technique, we were able to produce free-floating partly fluid-filled 3D-E spheroids in suspension. The brush biopsy did not require analgesics. Due to the minimal pain by a brush biopsy, repeated sampling could be offered a patient anytime needed.
- Few laboratory resources are needed. The yield of cells from a plain brush biopsy was sufficient to produce spheroids. No enzymatic splitting or washing steps were involved, and hence no cell loss due to such procedures.
- We found the 3D-E spheroids to be highly discriminative between PCD and healthy controls: the 3D-E spheroids separated equally well between PCD and healthy controls as compared to the initial cCFA, both by CBF and CBP.
- The validity of 3D-E spheroid CBP and CBF was high in both PCD and healthy controls, as CBP and CBF values did not differ significantly from their paired initial values found by our standard reference method (cCFA).
- The success rate was high. We had an overall success rate of 82%, which is superior to those previously described from other in vitro methods (54–79%) [6, 19].

The spheroids formed equally fast in PCD patients and in healthy controls. The reported difference in median harvesting time was due to coincidence. If nasal brushing had been performed on a Thursday or Friday, the spheroids would form during the following weekend, but not harvested until Monday.

Formation of 3D-E spheroids may be used as a much faster alternative to improve the diagnostic accuracy in diagnosing of PCD in cases where conventional work-up shows to be difficult. Our 3D-E spheroids showed valid ciliary function proven by CBP and CBF in at least 5 days in culture, and vital cilia proven by beating still after 16 days. This makes it possible to re-analyze ciliary function from the same patient’s sample over an expanded time period if needed, in the same way as can be done by other in vitro methods [7, 8]. We therefore believe that our 3D-E spheroid model may be useful for further understanding the pathophysiology of PCD and parameters of airway epithelium in general.

We further showed it feasible to characterize the 3D-E spheroids by IHC analysis. In line with previous observations [9–12], our analysis shows that the spheroids present well-organized arrangements of monolayer cells enclosing a partly fluid-filled lumen with the ciliated apical surface facing the outside. Further, IHC of the spheroids may come into hand for in-depth analyses of spheroids derived from healthy individuals versus PCD patients in relation to identification of axonemal aberrations as well as defects in ciliary signaling systems that impact on ciliary beating.

In our 3D-E spheroid model, we used nasal cells from minimally invasive brush biopsies. This was different from the previous reported 3D-E model by Pedersen and co-workers [1, 9–12] and advantageous for us, since we did not need to rely on a patient having polyps for getting material and since we could skip all enzymatic steps otherwise needed to release the epithelial cells from the underlying polyp tissue. The yield of spheroids was less from a brush biopsy compared to the yield from a whole nasal polyp.

### Limitations of the study

Our study was small and among only eight cases of successful PCD spheroids, we had four different types of dysmotility. More samples would be required in future studies to prove that our method is able to consistently replicate all dysmotilities among PCD patients.

In many cases, but not always, healthy cells formed rotating spheroids. Again, due to limited number of samples, a consistent pattern was difficult to find. It seemed that healthy cells aggregating in large spheroids were more prone to form rotating spheroids, since we exclusively saw large rotating spheroids. In future, larger studies will be needed to further describe this phenomenon as well as more detailed quantitative and qualitative analyses of the spheroid cilia.

With time, the spheroid cells will die. Day-to-day assessment of the fraction of living and dead ciliated cells in the spheroids was not performed in this study. However, the spheroids were harvested after only a few days (1–5 days) and CBF as well as CBP was unchanged, when comparing spheroid CBF and CBP to the initial cCFA CBF and CBP assessments. Thus suggesting that the overall ciliary functionality was preserved in the spheroids at the time they were harvested.

We only assessed CBP and CBF by high-speed analysis at a maximal spheroid age of 5 days in culture. A single batch was kept with live and beating cilia for 16 days. Further studies are needed to determine for exactly how long the 3D-E spheroid cells maintain their initial ciliary functionality, when produced by the presented method. In a previous study of 3D-E spheroids produced from lung tissue and nasal polyps, the spheroids were kept viable for 6 weeks when derived from lung tissue and up to 6 months when derived from nasal polyps [20].

PCD patients are chronically infected in the nose. By adding antibiotics (penicillin/streptomycin) to the cell medium, the risk of losing spheroids due to infection is presumably less. Still, we lost 3/11 PCD samples to infection, compared to no spheroid infections in our healthy group. However, our overall success rate of 82% was still superior to previous reports (54–79%) [6, 19].

Spheroid CBP and CBF assessment required immobilization of the spheroids.

Examining free moving spheroids without destroying their form would allow better options for further assessing movements of these spheroids, including those spinning around their own axis (example shown in Additional file 1). However, at this point we do not have a solution for this challenge.

## Conclusion

We have shown three-dimensional explant (3D-E) spheroid formation from patient-specific minimal invasive nasal brush biopsy to be an extremely fast and easy ex vivo method for investigating ciliary function in airway epithelium, including a potential for assisting the diagnosis of PCD and with further potential for investigating pathophysiological aspects of ciliated epithelium and of PCD.

## Additional files

**Additional file 1.** Rotating healthy 3D-E spheroid. Recorded at a frame rate of 83 frames per second.

**Additional file 2.** Non-rotating healthy 3D-E spheroid. Recorded at a frame rate of 83 frames per second.

**Additional file 3.** 3D-E spheroid from a patient with PCD (outer dynein arm deficiency). Recorded at a frame rate of 83 frames per second.

## Abbreviations

ALI: air–liquid interface
cCFA: conventional ciliary function analysis
3D-E: three-dimensional explant
CBF: ciliary beat frequency
CBP: ciliary beat pattern
DIC: differential interference contrast
fl: partly fluid-filled lumen
HSVA: high-speed video analysis
ICC: immunocytochemical analysis
IDA: inner dynein arm
IF: immunoflourescence
IHC: immunohistochemistry
nNO: nasal nitric oxide
ODA: outer dynein arm
PCD: primary ciliary dyskinesia
